# Hypolimnetic assimilation of ammonium by the nuisance alga *Gonyostomum semen*

**DOI:** 10.3934/microbiol.2020006

**Published:** 2020-04-22

**Authors:** Thomas Rohrlack

**Affiliations:** Norwegian University of Life Sciences, Faculty for Environmental Sciences and Natural Resource Management, Postbox 5003, NO-1432 Ås, Norway

**Keywords:** *Gonyostomum semen*, hypolimnetic ammonium uptake, algal blooms, harmful algae, nutrient recycling

## Abstract

*Gonyostomum semen* is a bloom-forming freshwater raphidophyte that is currently on the increase, which concerns water managers and ecologists alike. Much indicates that the recent success of *G. semen* is linked to its diel vertical migration (DVM), which helps to overcome the spatial separation of optimal light conditions for photosynthesis at the surface of a lake and the high concentration of phosphate in the hypolimnion. I here present data from a field study conducted in Lake Lundebyvannet (Norway) in 2017–2019 that are consistent with the idea that the DVM of *G. semen* also allows for a hypolimnetic uptake of ammonium. As expected, microbial mineralization of organic matter in a low-oxygen environment led to an accumulation of ammonium in the hypolimnion as long as *G. semen* was absent. In contrast, a decreasing or constantly lower concentration of hypolimnetic ammonium was found in presence of a migrating *G. semen* population. In summer of 2019, a short break in the DVM of *G. semen* coincided with a rapid accumulation of hypolimnetic ammonium, which was equally rapidly decimated when *G. semen* resumed its DVM. Taken together, these data support the idea that *G. semen* can exploit the hypolimnetic pool of ammonium, which may be one reason for the recent success of the species and its significant impact on the structure of the aquatic food web.

## 1. Introduction

*Gonyostomum semen* is a freshwater raphidophyte with a global distribution [1]–[5]. The species is currently undergoing a marked expansion in Fennoscandia and the Baltic countries, where it forms dense blooms in an increasing number of lakes [5]–[12]. This development is of concern to water managers and ecologists alike. The species excretes slimy filaments that can irritate the human skin and that may interfere with drinking water production by blocking filters [7],[10],[13]. The dominance of *G. semen* has also been linked to significant changes in the aquatic food web, including an increase in the relative importance of heterotrophic microbes [14], a decline of large-bodied, herbivorous zooplankton species [15],[16] and changes in the species composition and age structure of the fish fauna [17].

Much indicates that the recent success of *G. semen* is linked to its diel vertical migration (DVM). Several studies found that the species migrates into the hypolimnion in the evening, where it spends the night taking up phosphate [3],[10],[18]. A recent study even showed that in shallow lakes *G. semen* forms a layer on top of the sediment at night, which gives the species direct access to a virtually unlimited source of phosphate [19]. The second component of the DVM, the migration towards the surface in the morning, may allow *G. semen* to seek depths with light conditions optimal for photosynthesis [20]. This explains why the species is highly successful in boreal lakes, where other phytoplankton is inhibited by the shading effect of humic substances [6],[8],[9],[21],[22]. Taken together, these findings suggest that *G. semen* escapes phosphorus and light limitation as long as its DVM bridges the distance between depths with optimal light conditions and those with an adequate supply of phosphate.

The next logic question is then whether the DVM also gives access to the hypolimnetic pool of dissolved inorganic nitrogen as suspected by some authors [10],[18] and as it is common in many other migrating phytoplankton species. *Chattonella antiqua*, a marine relative of *G. semen*, for example, migrates to the hypolimnion at night where it assimilates nitrate [23]. Similar results were reported for dinoflagellates [24],[25]. However, this nocturnal nitrate assimilation may be inhibited by the presence of high ammonium concentrations [26], and it may depend on a sufficient light supply during the preceding day because algae require energy to reduce nitrate to ammonium [27]. Moreover, the hypolimnion of lakes with *G. semen* blooms is often anoxic for longer periods of time [10],[18], causing denitrification of nitrate and most likely an accumulation of ammonium. It is therefore reasonable to assume that the capacity to assimilate hypolimnetic ammonium would be of more advantage to *G. semen* than a hypolimnetic uptake of nitrate [28],[29].

The present study tested the hypothesis that *G. semen* forms blooms by exploiting the hypolimnetic pool of ammonium. The study built on the generally accepted theory that lakes with a high concentration of dissolved organic matter (DOM) are dominated by heterotrophic processes [21],[30]–[33], which should continuously release ammonium into the hypolimnion. The decomposition of biomass formed during *G. semen* blooms should further intensify the production of ammonium in the hypolimnion. The mineralization of DOM and *G. semen* biomass should also result in a rapid depletion of hypolimnetic oxygen, which, in turn, should prevent nitrification, i.e., the microbial oxidation of ammonium. Under these conditions, one expects to see an accumulation of ammonium in the hypolimnion. A lack of this accumulation or even a reduction in hypolimnetic ammonium coinciding with the presence of migrating *G. semen* would then suggest the occurrence of a hypolimnetic assimilation of ammonium. That *G. semen* can assimilate ammonium at all appears certain since ammonium is considered the most preferred nitrogen source for phytoplankton in natural waters [34].

## 2. Material and methods

### 2.1. Study area

The study focused on Lake Lundebyvannet, a shallow (maximal depth 5.3 m) and comparatively small water body (surface area of 0.4 km^2^), which is situated about 50 km southeast of the city of Oslo (Norway). The lake receives large amounts of allochthonous DOM but is also rich in autochthonous DOM [35]. A water color of more than 100 mg Pt L^−1^ [6] limit light penetration to the upper 2–3 m of the water column, resulting in steep temperature gradients and thermal stratification during summer. On average, the hypolimnion covers the lower two meters of the water column. The lake has a long history of *G. semen* blooms [36] that typically last from May/June to September. A recent study found that the DVM of the local *G. semen* population shows a surprisingly consistent pattern with a upwards migration that is synchronized with sunrise and a downwards migration that is initiated the earlier the warmer the water column is [19]. The phytoplankton of Lake Lundebyvannet is completely dominated by *G. semen*. Other species occur only in traces (public access to monitoring data via https://vannmiljo.miljodirektoratet.no/).

### 2.2. Field work

The lake was sampled throughout three seasons (2017–2019). Field work of a given season was initiated after the lake became free of ice and it was concluded when the onset of stormy autumn weather ended the *G. semen* bloom. Samples were taken at the deepest point of the lake (depth: 5 m, geographic position: 59.54911 N, 11.47843 E) at 3–14 days intervals. Integrated samples from the epilimnion (upper three meters of the water column) and from the hypolimnion (lower two meters of the water column) were taken at 10 a.m., i.e., hours after the arrival of *G. semen* in the epilimnion on a typical day [19]. Subsamples for nitrate and ammonium analyses were filtered through 0.45 µm cellulose acetate membrane filters immediately after sampling and were stored frozen at -21 °C. Dissolved ammonium and nitrate were quantified according to ISO standard methods 7150-1:1984 and ISO 10304-1:2007. Subsamples from the epilimnion for pigment analysis were filtered through 45 mm GF/C glass fiber filters, which were then placed in 15 ml plastic tubes to be stored at -21 °C. The oxygen saturation at 4 and 5 m was measured using a pre-calibrated dissolved oxygen probe.

The temperature profile was recorded with a chain of HOBO temperature sensors (UA-002-64, Onset Computer Corporation, Bourne, USA), set to log data every 2.5–10 min. The occurrence of *G. semen*'s DVM was monitored with a self-cleaning chlorophyll *a* probe (YSI 6025, YSI Incorporated, Yellow Springs, USA), set to log data every 2.5–10 min. The probe was placed 1 m (2017/2018) or 2 m (2019) beneath the surface of the lake. The DVM of *G. semen* revealed itself by recurring shifts between high chlorophyll readings during the day and near zero readings during the night.

### 2.3. Abundance of *G. semen*

The species produces the rare xanthophyll heteroxanthin that served as biomass marker to estimate the abuandance of *G. semen* [36]. Pigments were extracted from freshly lyophilized filters by adding 3 ml acetone to each filter and by keeping the samples at 4 °C overnight. The extracts were centrifuged at 2000 x g for 10 minutes to remove particles.

Pigment analysis was done by HPLC immediately after extraction to avoid post-extraction derivatization. The instrumental setup included a Thermo Fisher Ultimate 3000 UHPLC RS system equipped with diode array detector and an Acclaim C30 LC column (150 x 2.1 mm, 3 µm particle size). The entire hardware was supplied by Nerliens Meszansky AS (Oslo, Norway). Pigment separation and identification followed the method described by Wright and coworkers [37], except for the flow rate of the HPLC pump that had to be reduced to 0.5 ml min^−1^ to match the specifications of the C30 LC column. Pigments were identified on the basis of their retention times and absorption spectra (350–700 nm).

There is no commercial standard for heteroxanthin. I therefore used the peak area ratio between heteroxanthin and chlorophyll a given by Hagman et al. [36] (heteroxanthin/chlorophyll a = 0.045) to estimate the amount of *G. semen*-derived chlorophyll a in the water column. The effect of the abuandance of *G. semen* on the concentration of ammonium in the hypolimnion was studied by running Spearman rank correlation tests for periods with *G. semen* occurrence.

## 3. Results

### 3.1. General environmental conditions

**Figure 1.**
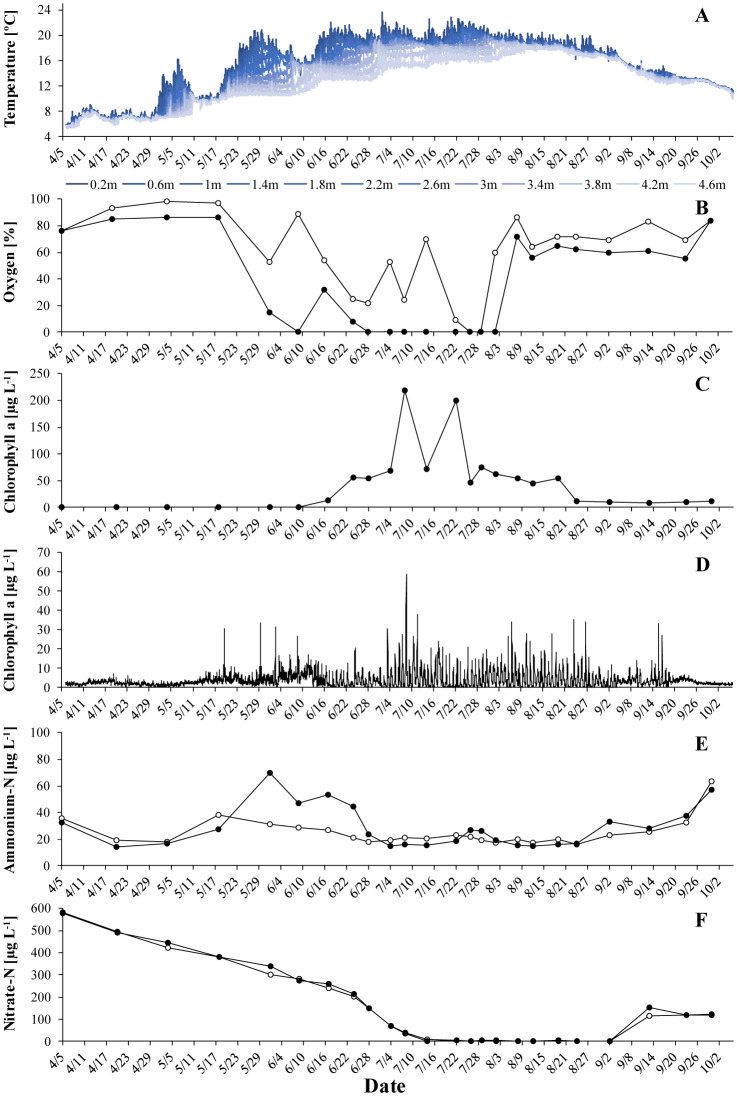
Situation in Lake Lundebyvannet in 2017: A: temperature profile, B: oxygen saturation at 4 m (open circles) and 5 m (black circles), C: mean concentration of *G. semen*-derived chlorophyll a in the epilimnion, D: signal of the chlorophyll a sensor at 1 m (when comparing data shown in panel C and D please keep in mind that due to an uneven depth-distribution of *G. semen* the mean concentration of chlorophyll a in the epilimnion can be larger than the concentration at 1 m, E: concentration of ammonium in the epilimnion (open circles) and hypolimnion (black circles), F: concentrations of nitrate in the epilimnion (open circles) and hypolimnion (black circles).

**Figure 2.**
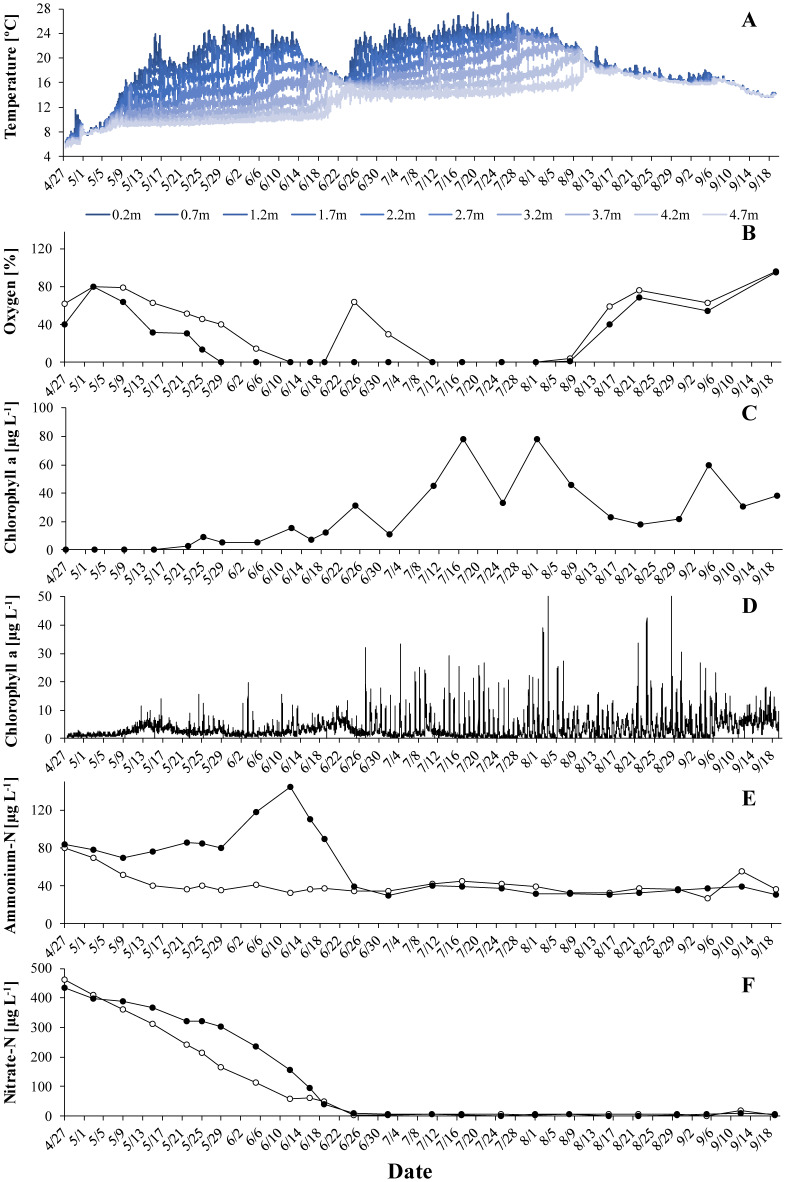
The situation in Lake Lundebyvannet in 2018: A: temperature profile, B: oxygen saturation at 4 m (open circles) and 5 m (black circles), C: mean concentration of *G. semen*-derived chlorophyll a in the epilimnion, D: signal of the chlorophyll a sensor at 1 m, E: concentration of ammonium in the epilimnion (open circles) and hypolimnion (black circles), F: concentration of nitrate in the epilimnion (open circles) and hypolimnion (black circles).

**Figure 3.**
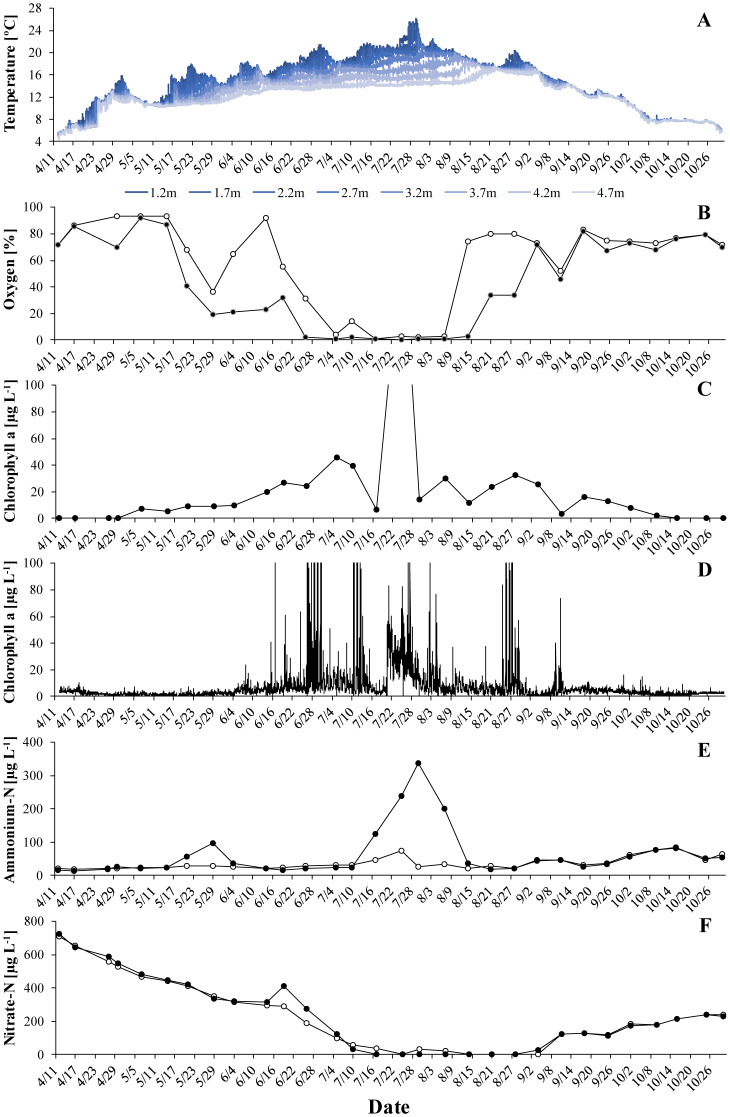
The situation in Lake Lundebyvannet in 2019: A: temperature profile, B: oxygen saturation at 4 m (open circles) and 5 m (black circles), C: mean concentration of *G. semen*-derived chlorophyll a in the epilimnion. The largest peak (213 µg L^−1^) was truncated to better show smaller values. D: signal of the chlorophyll a sensor at 2 m, E: concentration of ammonium in the epilimnion (open circles) and hypolimnion (black circles), F: concentration of nitrate in the epilimnion (open circles) and hypolimnion (black circles).

**Figure 4.**
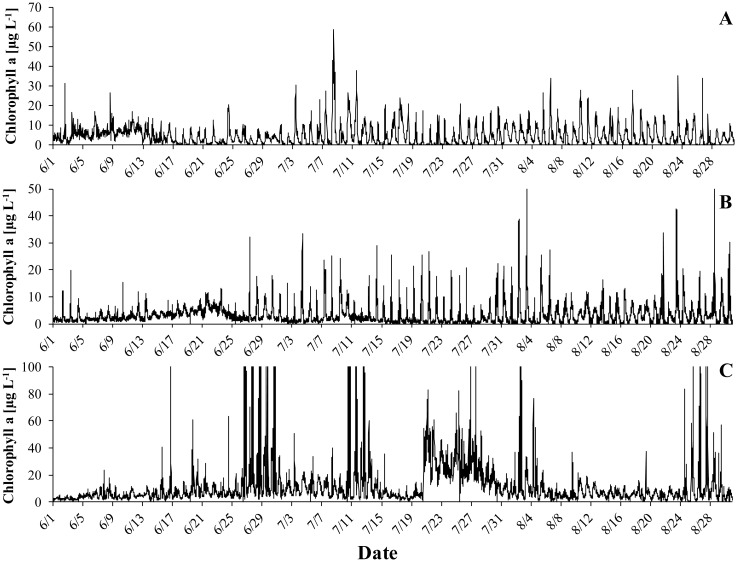
This figure intends to demonstrate the DVM of *G. semen* more clearly by showing the data of Figures 1D–3D (output of the chlorophyll a sensor) for the periods with high *G. semen* abundances (A: 2017, B: 2018, C: 2019).

Thermal stratification typically lasted from May to mid-August (Figures 1A–3A) and coincided with oxygen depletion in the hypolimnion (Figures 1B–3B). In all years, short periods with anoxic conditions were observed at 4 m. At 5 m, the water was anoxic for major parts of the period with thermal stratification. The longest such period was observed in 2018, when dissolved oxygen was undetectable from end of May to the beginning of August. *G. semen* appeared in May or June (Figures 1C–3C). The highest abundances were observed in July and the beginning of August. The presence of *G. semen* was accompanied by a typical diurnal pattern of the chlorophyll a probe signal, with high readings during the day and near-zero readings during the night (Figures 1D–3D, Figure 4). This indicated that *G. semen* continuously conducted its DVM. However, the situation was different between July 21 and 30, 2019, when high chlorophyll a probe readings during day and night indicated that *G. semen* had discontinued its DVM. During periods with high *G. semen* abundances, the concentration of ammonium in the epilimnion was between 10 and 40 µg N L^−1^ (Figures 1E–3E) and, in many cases, nitrate could not be detected throughout the water column (Figures 1F–3F).

### 3.2. Indications of hypolimnetic assimilation of ammonium by *G. semen*

In 2017, the occurrence of low oxygen levels at 5 m led, as expected, to an accumulation of hypolimnetic ammonium (Figures 1B and 1E). Shortly after, the concentration of ammonium in the hypolimnion dropped. This drop coincided with a rapid increase in the abundance of *G. semen* and a shift in the chlorophyll a probe signal towards a pattern indicating that *G. semen* performed its DVM (Figures 1C–E). Here it is important to mention that the drop in hypolimnetic ammonium happened while the water column was stably stratified (Figure 1A), meaning that the loss of ammonium from the hypolimnion could not have been due to vertical transport of water. The remainder of the period with thermal stratification was characterized by high concentrations of *G. semen* and a chlorophyll a probe signal typical for the DVM of *G. semen*. Hypolimnetic ammonium remained at a constantly low level although the lack of oxygen at 5 m should have prevented the loss of ammonium due to nitrification. Very similar observations were made in 2018 (Figure 2). For both years, a negative correlation was found between the abundance of *G. semen* and the concentration of ammonium in the hypolimnion (2017: r = -0.49, p < 0.05; 2018: r = -0.53, p < 0.05).

No such correlation was found in 2019. Instead, an interesting incident was witnessed in summer 2019. Both, the chlorophyll a probe and the pigment analysis returned low values in mid-July (Figures 3C and 3D, Figure 4C). Shortly after, the abundance of *G. semen* increased again but constantly high chlorophyll a probe readings showed that the species stayed in the epilimnion also during the night. Both periods coincided with a strong increase in the concentration of hypolimnetic ammonium (Figure 3E). On August 1, the reappearance of the diurnal rhythm of the chlorophyll a probe signal indicated that *G. semen* had reestablished its DVM. This coincided with a rapid decrease in hypolimnetic ammonium, which happened during a period without oxygen for nitrification at 5 m.

## 4. Discussion

Data from Lake Lundebyvannet support the idea that *G. semen* can assimilate ammonium from the hypolimnion. Prior to the occurrence of high *G. semen* densities, thermal stratification and low oxygen values led to an accumulation of ammonium in the hypolimnion of Lake Lundebyvannet. Hence, without *G. semen* the processes which supplied ammonium to the hypolimnion outpaced those that eliminated ammonium. In contrast, the occurrence of high densities of migrating *G. semen* were always associated with a decrease in hypolimnetic ammonium or a stably low concentration of the nutrient, suggesting that the species indeed assimilated ammonium during its nocturnal stay in the hypolimnion. This was most obvious in July 2019, when a break in the DVM of *G. semen* coincided with a built-up of hypolimnetic ammonium, which was rapidly decimated when *G. semen* re-established its DVM.

Data of the present study also underline the adaptive value of a hypolimnetic assimilation of ammonium. The half-saturation constant of ammonium uptake by phytoplankton ranges from 1.4 to 80 µg N L^−1^ [38] and that of *Chattonella antiqua*, a close relative of *G. semen*, is 31 µg N L^−1^ [39],[40]. The half-saturation constant for phytoplankton growth is even higher, ranging from 35 to more than 400 µg N L^−1^ [41],[42]. In Lake Lundebyvannet, the concentration of ammonium in the epilimnion was between 10 and 40 µg N L^−1^ during periods with high *G. semen* abundances and there was often no detectable nitrate in the water column. This implies that non-migrating phytoplankton species would have been nitrogen-limited. The assimilation of ammonium from the hypolimnion of Lake Lundebyvannet may have allowed *G. semen* to avoid or to reduce nitrogen limitation. It must also be taken into consideration that algal growth and the sedimentation of dead algal cells cause a net-transport of nitrogen from the epilimnion into the hypolimnion, where mineralization constantly refills the local pool of ammonium. The capacity to assimilate hypolimnetic ammonium therefore ensures access to a permanently available source of inorganic nitrogen.

The ability to exploit the hypolimnetic pool of ammonium has significant implications for the ecology of *G. semen*. An obvious consequence is that in shallow lake the species may live exclusively on the ammonium that continuously leaks from the sediment into the hypolimnion. This may allow for bloom formation in lakes with a nitrogen-depleted epilimnion, which may explain why *G. semen* is particularly successful in shallow lakes [1]. The hypolimnetic ammonium uptake may also contribute to the recycling of nutrients during a bloom. *G. semen* has relatively large cells and can expel long, slimy filaments, the so-called trichocysts, upon mechanical stimulation [10]. Both features may limit the grazing by filter-feeding herbivorous zooplankton [16],[43]. The impact of zooplankton may be further reduced by the DVM of *G. semen* that is inverse to that of many zooplankton species, which reduces the probability of an encounter between the alga and its potential enemies [18]. Nevertheless, the density of *G. semen* in Lake Lundebyvannet showed considerable fluctuations over a season (Figures 1C–3C), suggesting that at times large amounts of biomass were lost. If this biomass was not ingested by zooplankton, it was probably broken down by microbes in the hypolimnion or at the surface of the sediment. This microbial mineralization would have released ammonium into the hypolimnion, where it may have been assimilated by *G. semen* as suggested above. The same may have happened with the phosphate that was released during mineralization of *G. semen* biomass [18].

Such a recycling of nutrients would deprive less motile phytoplankton species of growth resources, because nutrients are assimilated by *G. semen* before they can be transported to the upper part of the water column by diffusion or turbulence. This may explain why *G. semen* under optimal conditions forms nearly unialgal blooms [2],[17],[19],[44]. The lack of edible phytoplankton may then lead to the often observed shift from large filter-feeding zooplankton to small species that live on bacteria [2],[15],[16] and to the also well-documented secondary effects of *G. semen* dominance on higher trophic levels [17]. We may thus assume that the hypolimnetic uptake of ammonium is one of the reasons for that the occurrence of *G. semen* blooms leads to a restructuring of the aquatic food web [14],[15],[17].

The results of the present study also underline the challenge that *G. semen* poses to water managers but also to ecologists. The species is certainly undergoing an expansion that threatens human interests and the integrity of aquatic ecosystems [5]–[12]. But how should water management mitigate the impact of a species that, owing to its DVM, has access to optimal light conditions [20], phosphorus [18] and, as shown here, nitrogen from the hypolimnion or maybe even from the sediment surface [19], where the microbial mineralization of organic matter continuously supplies phosphate and ammonium. And if *G. semen* under optimal conditions escapes light, phosphorus and nitrogen limitation as well as zooplankton grazing, what was causing the fluctuations in *G. semen* density that were observed in Lake Lundebyvannet each summer? One option is that the built-up of high *G. semen* densities caused inorganic carbon limitation of photosynthesis, which is known to be a source of metabolic stress in for example cyanobacterial blooms [45]. However, the occurrence of carbon limitation would be in striking contradiction to the generally accepted theory that humic lakes such as Lake Lundebyvannet are supersaturated with CO_2_ [21],[46]–[48].

Over time, many factors have been suggested to contribute to the success of *G. semen*, including its access to phosphate from the hypolimnion or sediment and to optimal light conditions near the surface [3],[10],[18],[20], its grazing resistance [16], as well as its promotion by humic substances, lake browning [2],[6],[7],[9],[10],[49], global warming [8], and a high concentration of iron [50]. The present study added the hypolimnetic uptake of ammonium to the list, which, as many of the other factors favoring *G. semen*, either promote the species' DVM or result from it. This underlines the necessity to better understand this migration.
